# Exploration of motion inhibition for the suppression of false positives in biologically inspired small target detection algorithms from a moving platform

**DOI:** 10.1007/s00422-022-00950-9

**Published:** 2022-10-28

**Authors:** Aaron Melville-Smith, Anthony Finn, Muhammad Uzair, Russell S. A. Brinkworth

**Affiliations:** 1 Defense and Systems Institute, UniSA STEM, University of South Australia, Adelaide, SA 5095 Australia; 2 College of Science and Engineering, Flinders University, Tonsley, SA 5042 Australia

**Keywords:** Biological vision, Target detection, Saliency enhancement, Optic flow, 92-10, 92-08

## Abstract

Detecting small moving targets against a cluttered background in visual data is a challenging task. The main problems include spatio-temporal target contrast enhancement, background suppression and accurate target segmentation. When targets are at great distances from a non-stationary camera, the difficulty of these challenges increases. In such cases the moving camera can introduce large spatial changes between frames which may cause issues in temporal algorithms; furthermore targets can approach a single pixel, thereby affecting spatial methods. Previous literature has shown that biologically inspired methods, based on the vision systems of insects, are robust to such conditions. It has also been shown that the use of divisive optic-flow inhibition with these methods enhances the detectability of small targets. However, the location within the visual pathway the inhibition should be applied was ambiguous. In this paper, we investigated the tunings of some of the optic-flow filters and use of a nonlinear transform on the optic-flow signal to modify motion responses for the purpose of suppressing false positives and enhancing small target detection. Additionally, we looked at multiple locations within the biologically inspired vision (BIV) algorithm where inhibition could further enhance detection performance, and look at driving the nonlinear transform with a global motion estimate. To get a better understanding of how the BIV algorithm performs, we compared to other state-of-the-art target detection algorithms, and look at how their performance can be enhanced with the optic-flow inhibition. Our explicit use of the nonlinear inhibition allows for the incorporation of a wider dynamic range of inhibiting signals, along with spatio-temporal filter refinement, which further increases target-background discrimination in the presence of camera motion. Extensive experiments shows that our proposed approach achieves an improvement of 25% over linearly conditioned inhibition schemes and 2.33 times the detection performance of the BIV model without inhibition. Moreover, our approach achieves between 10 and 104 times better detection performance compared to any conventional state-of-the-art moving object detection algorithm applied to the same, highly cluttered and moving scenes. Applying the nonlinear inhibition to other algorithms showed that their performance can be increased by up to 22 times. These findings show that the application of optic-flow- based signal suppression should be applied to enhance target detection from moving platforms. Furthermore, they indicate where best to look for evidence of such signals within the insect brain.

**Supplementary Information:**

The online version contains supplementary material available at 10.1007/s00422-022-00950-9.

## Introduction

Small target detection in visual scenes has attracted significant research attention owing to its applications in a wide range of areas such as search and track (Gao et al. 2013), surveillance (Butler 2008), defence (Chen et al. 2014) and collision mitigation systems (Perry 1997; Li et al. 2016a). Electro-optic and infrared cameras are often used for such applications as they offer a cost effective, small and lightweight option. Long distances between the sensor and targets can mean the objects of interest may only occupy a few pixels in the image (pixel sized targets), with no shape or texture information cues to help extract them (Gao et al. 2013). Couple this with atmospheric effects and low signal to clutter ratios due to clouds, water ripple and trees, and the task of successful detection with minimal false alarms becomes extremely challenging (Xie et al. 2014).

A variety of conventional computer vision approaches exist in the literature for detecting moving objects against cluttered environments (Sobral and Vacavant 2014; Xu et al. 2016; Zhao et al. 2019; Li et al. 2016b). These methods were mostly designed for detecting large objects (such as humans, animals, cars, etc.) that generally occupy several hundred pixels within the image. Moreover, these methods heavily rely on well defined shape, colour and textural features to build their object detection models. In contrast, the spatial resolution of the pixel sized targets that are studied in this paper ranges from a few pixels to a single pixel without any shape or textural cues. Such targets are very hard to visually discriminate from sensor noise and cluttered background features. Conventional object detection methods do not take into account these challenges and may completely fail when applied to the problem of pixel sized target detection. Similarly, state-of-the-art neural network approaches often require larger targets as they are biased towards texture (Geirhos et al. 2018), for which there is none for such small targets. This can cause neural networks to perform poorly as they either miss targets or produce a high number of false detections (Gao et al. 2018).

Through millions of years of evolution, the visual system of many species of small flying insects has perfected an astounding capability to detect and track small moving targets in cluttered backgrounds (Pritchard 1965; O’Carroll 1993; Olberg et al. 2000; Nordström et al. 2006). Due to their relatively simple structure and small size, the visual pathway of small flying insects has been investigated and computationally modelled in different studies over the last few decades (Hassenstein and Reichardt (1956a); Arnett 1972; Payne and Howard 1981; Hardie and Weckström 1990; Jansonius and Van Hateren 1991; Osorio 1991; Van Hateren and Snippe 2001; Higgins and Pant 2004; Van Hateren and Snippe 2006). One biologically inspired vision (BIV) model (Wiederman et al. 2008a, b, c, 2010) built upon these studies has been shown to be extremely robust to the challenges of small target detection against cluttered backgrounds in natural scenes. The multi-stage BIV has also been shown recently to significantly outperform state-of-the-art conventional small target detection and tracking methods (Bagheri et al. 2017; Melville-Smith et al. 2019).

A practical, but extremely challenging, scenario for small target detection is when the targets are far away and the scene is captured by a camera mounted on a moving platform such as a robot, aircraft or drone. The biological visual systems of small flying insects deal with ego motion robustly (Wertz et al. 2009). The motion pathways of the BIV, which have been modelled on those found in insects have been shown to be advantageous for rotational velocity estimations (Skelton et al. 2019) and enhancing the saliency of targets (Wiederman et al. 2008b).

For algorithms with a temporal component, we observe that when such motion is induced onto imaging sensors, the temporal filter responses can often create more false positives in areas of clutter. For algorithms that only have a spatial component, performance is often independent of ego-motion characteristics; however, many false positives can still occur in regions of clutter. Wiederman et al. showed that the motion estimation pathway of the BIV can provide an output that is related to temporal changes in local contrast and is a good estimator to identify regions of clutter. It’s use as an inhibitor on the models target saliency output showed benefit, increasing the separation of small targets from the background. In this paper, we expand upon the work of Wiederman et al. , which exploits low level scene motion features through optic-flow, by investigating whether there are better performing, and potentially more biologically plausible, locations earlier in the model to implement a motion inhibition mechanism, rather than at the location presented by Wiederman et al. (the models output). The possible key stages are selected based on our careful examination of their responses to the inhibition signal under simulated camera motion. Additionally, we also look at adding a new layer of nonlinear conditioning to the motion inhibition signal as well as tuning some of the optic flow filters specifically for the purpose of small target detection. Our explicit use of a compressive nonlinearity allows for the incorporation of a wider dynamic range in the inhibiting signal along with spatio-temporal refinement which further increases target-background discrimination in the presence of camera motion. Finally, we look at using the conditioned motion signal and apply it as an inhibitor to the output of other algorithms to see how their performance can also be improved.

### Comparative small target detectors

Background subtraction methods are often used to find larger moving objects within an environment; however when large amounts of motion are induced by a moving platform, performance can degrade (Garcia-Garcia et al. 2020). The detection of small targets is possible with methods such as the pixel-based adaptive segmenter (PBAS) (Hofmann et al. 2012) in simulated scenarios which have static backgrounds (Melville-Smith et al. 2019), but when motion is induced on the background imagery, performance degrades significantly.

The local contrast method (LCM) (Chen et al. 2014) is an algorithm inspired by the human visual system (HVS) and designed for the detection of small, dim targets. Traditionally LCM has been used on thermal infrared imagery where target responses stand out from the background more than in the visible spectrum. The method measures dissimilarity between the current location and its neighbourhoods, thereby enhancing target signals while simultaneously suppressing background clutter. In testing it was shown to outperform top-hat (Tom et al. 1993) and the average grey absolute difference maximum map (Wang et al. 1995) methods for the purposes of small infrared target detection. Other research groups have taken inspiration from the LCM to create new algorithms, such as the spatial-temporal local contrast filter (STLC) (Deng et al. 2016), which calculates separate spatial and temporal contrasts and correlates them to find moving targets, and the multi-scale relative local contrast method (MRLCM) (Han et al. 2018) which looks at normalising the local contrast measures over multiple kernel sizes, rather than using an absolute contrast measure, and correlating each scale for a result. These methods have been shown to perform well; however, they assume that the background is mostly uniform, as is often the case with thermal infrared imagery. STLC makes the assumption that the camera is static, looking for changes in pixel intensity over time as the temporal component to detect targets. This can cause issues when the entire background is moving, as the assumptions made about the temporal and spatial correlation no longer hold true, causing many false alarms. The multi-scale aspect of MRLCM is seen as a disadvantage for this application, as all targets have a size that fits within the smallest kernel for the algorithm, 3$\times$3. Moving to larger scaled kernels is expected to have no advantage and reduce performance when the different scales are combined.

Taking inspiration from the many approaches that use the HVS, Xia et al. (2018) proposed a new target extraction method based on a local contrast measure combined with a modified random walker (MRW) algorithm. The output of the local contrast measure is used to generate a seed selection map from where the MRW algorithm begins segmenting the image into background and targets. This method outperformed other methods to which it was compared, including the multiscale patch-based contrast measure-based (MPCM) method (Wei et al. 2016), nonnegative infrared patch-image model based on partial sum minimisation of singular values-based (NIPPS) method (Dai et al. 2017), and local steering kernel (LSK) reconstruction-based method (Li and Zhang 2018). MRW was also found to have better background suppression than these methods, where high contrast edges, such as those often found around clouds and the horizon, cause false detections. This resulted in MRW being considered a more capable and robust method for finding targets in select environments.

Similarly, Qin et al. (2019) proposed a method similar to MRW based on a facet kernel and the random walker (FKRW) algorithm. This method first filters the imagery to remove pixel-sized noise with high brightness and then smooths the image using local order-statistic and mean filtering. This is done to facilitate the random walker algorithm, which performs better on images with less noise. A facet kernel, which is a kernel based on the facet model (Haralick 1987) used to find step edges, is then convolved with the image to enhance targets which are separated from the background through an adaptive threshold. Lastly, a novel local contrast descriptor based on the random walker algorithm is used to suppress clutter and further enhance target signals. The method has been shown to be more robust than other methods based on the HVS, such as LCM and its variants, over three scenes. This is due to FKRW’s ability to reduce background clusters which many other methods detect. It is suggested that the ability to reduce background clutter is due to the exploitation of directional consistency as a result of the facet kernel. Compared to the variable difference (VARD) (Nasiri and Chehresa 2017) algorithm, which is a method that compares the difference of the variance between three processed layers, background suppression appears to be similar, while FKRW was more robust over different scenes being able to detect the target more often. Compared to MRW, FKRW performed better when comparing ROC curve performance of true positives rates to false positives rates. FKRW was also found to be more efficient.

#### Biologically inspired vision (BIV) model

**Fig. 1:**
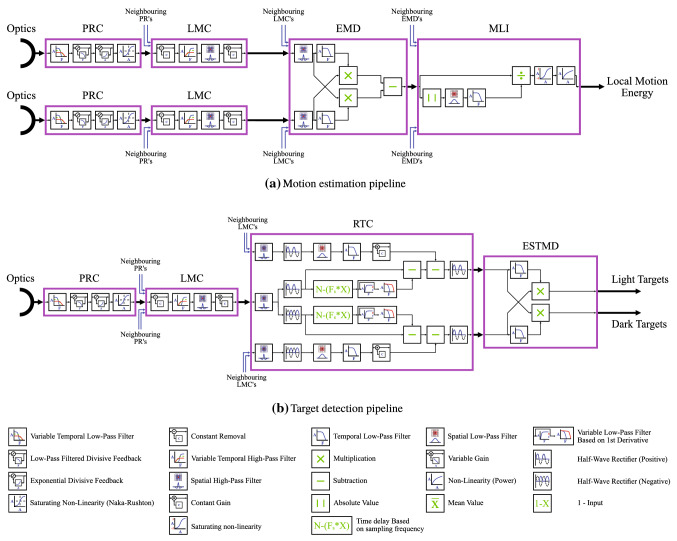
Illustration of the biologically inspired vision model (BIV) of the visual pathway of small flying insects. The original BIV model (Higgins and Pant 2004; Wiederman et al. 2008a, b, c; Brinkworth and O’Carroll 2009; Wiederman et al. 2010; Melville-Smith et al. 2019) offers two separate processing pipelines for motion estimation and target detection tasks. The small boxes represent the first order spatio-temporal filters and their combinations to realise the sequential processing mechanisms. The first two processing stages, the photoreceptor cells (PRC) and the lamina monopolar cells (LMC), are common to both pipelines. These stages perform spatio-temporal pre-processing of the raw input (the first two stages are represented twice in the diagram for the motion estimation pipeline as this requires input from two neighbouring pixels). The motion estimation pipeline models the elementary motion detection (EMD) cells and the medulla lobula interneuron (MLI) cell. The target detection pipeline models the rectifying transient cells (RTC) and the elementary small target motion detection (ESTMD) cells

Figure 1 shows the processing stages of the BIV model. The original BIV model has two separate processing pipelines for motion estimation and target detection tasks, where each pipeline processes the input image sequence independently.

The first two stages computationally model the insect photoreceptor cell (PRC) and lamina monopolar cell (LMC), both based off work by Van Hateren (Van Hateren 1992; Van Hateren and Snippe 2001), and are common to both pipelines. The PRC is used to enhance the signal to noise ratio (SNR) of the raw input image on a per-pixel level using variable low-pass filters controlled by each input pixel’s intensity (Griffiths 2018). Divisive and exponential feedbacks are used to produce fast and slow adaptation over time. Finally, a first order Naka-Rushton transform is used as a compressive nonlinearity to reduce the overall dynamic range of the signal. The LMC enhances important information, such as edges, while reducing redundant data (Van Hateren 1992). Both temporal and spatial elements have leaky high-pass filters applied, with the temporal domain being variably filtered on the pixel level based on the adaptation level from the PRC. Some models of the LMC have an additional nonlinearity (modelled by a tanh function) on the output. Biologically this makes sense, as it keeps the output signal within a fixed limit. However, it is not always necessary in a computer model without bandwidth limitations. This (optional) nonlinearity, and differing filter demands, explains why both follow-on processes in the BIV model have high-pass filters on their inputs when the LMC has one on its output. The PRC and LMC are powerful data pre-processors that together can also be used to enhance the performance of traditional target detection algorithms (Uzair et al. 2019, 2020a, b).

The motion estimation pipeline computationally models the elementary motion detection (EMD) cells, based on work by Hassenstein and Reichardt , and the medulla lobula interneuron (MLI) cells. While not physically modelled these stages have strong neurophysiological support for their existence (Hassenstein 1951; Hassenstein and Reichardt (1956a); Haag et al. 2004). From an engineering and mathematical modelling perspective the existence of the processing encapsulated by the elaborations to the basic EMD, and the entire MLI stage, are beneficial in reducing inter-scene variability in motion processing (Brinkworth and O’Carroll 2009). The EMD temporally correlates changes between neighbouring pixels to generate local optic flow vectors. These optic flow vectors are then normalised within the MLI using a nonlinear gain control to amplify the signal in regions of low clutter relative to regions of high clutter. Models designed to extract ego-motion have a subsequent processing stage based on the lobula plate tangential cells (LPTC) (Borst et al. 1995; Brinkworth and O’Carroll 2009; Borst et al. 2010; Skelton et al. 2019).

The target detection pipeline has two distinct outputs, one for bright targets and one for dark. Components of this pipeline include the rectifying transient cells (RTC), based on work by Jansonius and Van Hateren , and the elementary small target motion detector (ESTMD) neurons, based on a modified EMD. The RTC is one of the most important neurons in the target detection pipeline and, following electro-physiological recordings from fly brains, was originally modelled explicitly for this purpose (Wiederman et al. 2008c). It helps to enhance and separate falling and rising signals in time, such as those presented by small dark targets passing over a brighter background pixel. The input to the RTC is high-pass filtered and then two half-wave rectifiers are used to separate the positive and negative components of the signal. For each channel the derivative of each pixel is calculated over time to detect rising and falling signals. The rising signals induce a fast adaptation response while falling signals induce a slow response. The resulting signal is subtracted from the original half-wave rectified signal to negate periods where the signal continues to increase for long periods and to prevent multiple rapid detections. Such responses, if left unattenuated, can cause additional false detections, as well as target detections in both the light and dark output channels of the model: an unwanted result. To stop minor signals (which are unlikely to be targets) from triggering the fast adaptation, a threshold is used so that the derivative has to be above a defined value before the trigger comes into effect. To enact a fast response, a delayed signal is used to overcome sampling rate constraints of real digital sensor hardware. For falling signals, a low pass filter is used to give a slow adaptation from any previously detected rising signals, enforcing a refractory period between detections. To reduce the detection of larger objects or bars (high contrast lines), a local surround inhibition mechanism (Wiederman et al. 2008c) is used to suppress such features. The ESTMD is based on a theoretical model of the input to the small target motion detector (STMD) neuron  (O’Carroll 1993) and is not based on the actual neurophysiological recordings from within the fly brain. ESTMDs implement a modified elementary motion detector (Hassenstein and Reichardt 1956b) comparing the same point in space, rather than neighbouring spatial elements, across the two processed channels (rising and falling) from the RTC. The ESTMD takes the RTC output and temporally correlates rising and falling signals, which are often associated with small targets, on a per-pixel level. Essentially, the target detection pipeline will respond to two edges of opposite polarity in rapid succession. These signals of opposite polarity can exist in areas of high clutter or where transitions between background and foreground objects occur. In high clutter areas an increase in false positive detections can occur. This necessitates the need for a mechanism to suppress the false positives in these regions while maintaining true positive detection rates. Importantly, alternating rising and falling edges would also occur in regions of flicker. High-pass spatial filtering at the LMC (James 1992), as well as the presence of the surround inhibition within the RTC (Wiederman et al. 2008c), suppress responses to large-scale flicker, making the model respond much more strongly to spatially small targets.

For the models implementation used in this study, the filter time constants described in (Juusola et al. 1995; Van Hateren and Snippe 2001; Mah et al. 2006; Wiederman et al. 2008a, c; Brinkworth and O’Carroll 2009) were adapted for the simulations resolution, update rate (100 Hz), and background/target speed, where all corner frequencies used were below the Nyquist limit. The motion estimation pipeline has been shown to function in hardware at 100 frames/s (Skelton et al. 2019) and model parameters have been tuned using a genetic algorithm (Skelton et al. 2020).

#### Proposed nonlinear lateral inhibition scheme

**Fig. 2:**
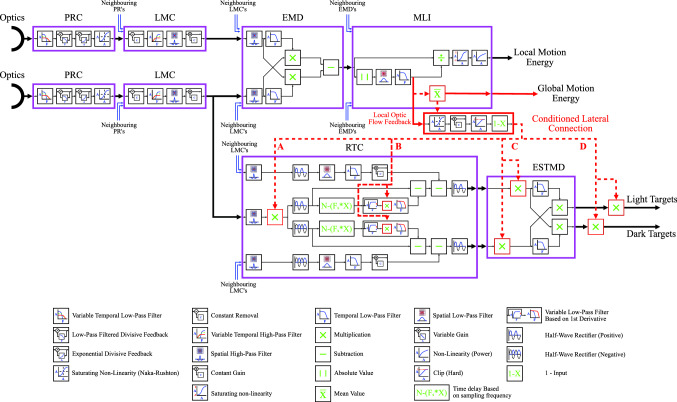
The camera motion adaptive biologically inspired vision model proposed in this paper. The proposed building blocks and key modifications to the model (relative to Fig. 1) have been highlighted in red. The local motion signal is taken from within the medulla lobula interneuron (MLI), conditioned, and then used to inhibit the signal at different locations in the small target detection model. The optional dynamic feedback is also shown, taking the mean from the local area motion and feeding it to the Naka-Rushton nonlinearity (note: the figure is best viewed in zoom mode)

Typically, when used for target detection, the BIV model only uses the PRC, LMC, RTC and ESTMD. However, previous work (Wiederman et al. 2008b) and a limited pilot study (Melville-Smith et al. 2019) showed that the use of a divisive inhibiting signal based on local motion from the addition of the EMD and MLI stages at locations **D** and **A**, respectively (see Fig. 2), was beneficial when ego-motion was induced into the imagery. Furthermore, it is known that using local motion adaptation during translational motion can improve the detected spatial structure within EMD-based models (Li et al. 2017). In Melville-Smith et al. (2019), not only were there a limited number of different environments tested but over-saturation of the feedback occurred when a linear conditioning was applied to the local area motion signal from within the MLI. While this suppressed false alarms it also suppressed the response of the system to real targets. Additionally, it was found that the inhibition calculations did not align with cluttered areas due to temporal filter parameters within the MLI, which caused many false positives (FP) to be detected on leading edges, and many true positives (TP) to be suppressed on trailing edges.

In this paper, we therefore propose a new nonlinear mechanism that further enhances the performance of small target detection. Our new contributions include: performing nonlinear conditioning on the lateral inhibiting signal from the absolute local-motion within the MLI, prior to the MLI local area normalisation and nonlinearities occurring, to reduce saturation; better tuning of the temporal low-pass filter within the MLI to create a more accurate feedback map; and testing multiple inhibition locations within the model to ascertain the location for best performance. We also examine the use of an inhibiting signal from an estimate of global ego-motion to condition the local area motion from the MLI dynamically. Finally, the performance of this newly proposed model was tested on a set of 20 diverse natural scenes. To discriminate between the various BIV models used in this paper, henceforth the original BIV model (Wiederman et al. 2008a) will be referred to as BIV ’08, the model with linear inhibition (Melville-Smith et al. 2019) as BIV ’19, and the nonlinear model presented in this paper as BIV ’22.

## Materials and methods

Figure 2 shows the modified BIV model with the new lateral connection linking the motion and target processing paths via nonlinear signal conditioning. Modifications are outlined in red with the tested inhibition locations denoted as **A**, **B**, **C**, and **D**. New and existing methods were tested using simulated data and their performance compared. The following sections outline the methodology for the simulations, model tuning and performance comparisons.

### Input imagery

To test the robustness of the algorithms to a variety of environmental settings 20 different real-world high dynamic range (HDR) panoramic environments were chosen. The HDR images ensured that the data being worked with was representative of the real-world environment without any quantisation or compression artefacts, which often occur in images designed for human viewing (a creative process), and exist in the majority of currently available datasets. This allows the BIV’s native information enhancing compression techniques of the PRC and LMC to be used to full effect.

The 20 natural images (having an intensity power and spatial frequency relationship of $\frac{1}{f^2}$ (Field 1987)) had varying structural differences. 14 of the images were published in (Brinkworth and O’Carroll 2007). The background images were created by stacking multiple exposures and mosaicing individual images into a panorama. The original panoramas were 8000 $\times$ 1600 pixels, covering 360$^\circ$ horizontal field of view and 72$^\circ$ vertical field of view. All image data was linear (no gamma or compression was applied) and each colour channel was stored in a 32bit floating point container.

**Fig. 3:**
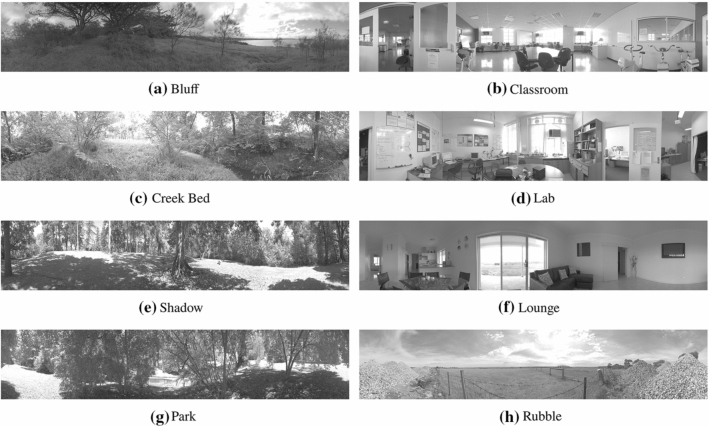
8 of the 20 high dynamic range panoramas used in this study. **a**–**f** were used to find an operating point for conditioning the inhibiting signal, MLI temporal filter and investigating the inhibition location. **g**, **h** are examples of test images with high and low levels of high frequency clutter, respectively. The images have been modified for ease of viewing and adapted from (Brinkworth and O’Carroll 2007). Only the green channel from each background was used to align with previous literature. The images selected for the tuning process covered multiple levels of clutter in order to find a general set of parameters for the BIV ’22 model

Each background had a different quantity of high-frequency spatial clutter, which is hypothesised to be one of the main effectors in pixel sized target detection. For this research, only the green channel of the panoramas was used as this closely represents the luminance in a scene and aligns with previous work. 6 of the backgrounds were used as a training set to find the best operating parameters for the feedback. These 6 backgrounds plus 2 others for reference can be seen in Fig. 3 (all 20 images used in this study can be seen in Online Resource 1).

### Target simulation

The background imagery was down-sampled to a size of $1000\times 200$ pixels to keep the simulation processing time manageable. 500 black squares (representing targets) were inserted at random locations onto the full sized backgrounds at a size that would occupy 1.2$\times$1.2 pixels after decimation. Minimum spacing between target centres was 10 pixels after decimation (3.6 degrees), a separation that has been shown not to cause significant cross-talk between target responses (Melville-Smith et al. 2019). Both the background and targets were animated separately with horizontal rotational motion in the same direction, moving right to left. The target and background rotational speeds tested on the down-sampled imagery were combinations of 10, 17, 29 and 50 pixels/s (corresponding to 3.6, 6.12, 10.44 and 18 degrees/s, or 0.1, 0.17, 0.29, and 0.5 pixels/frame) with a sampling frequency of 100 frames per second (FPS). The imagery then had a Gaussian blur applied so that when the imagery was decimated the full-width half-maximum (FWHM) was 1.0 pixels (initial testing showed this performed better than a FWHM of 1.4 pixels, as used previously (Wiederman et al. 2008a)). The imagery was then down-sampled using a nearest neighbour approach. While target speeds of 10 pixels/s is outside the tuned range of the model used here, we wanted to investigate if the operating range of the model could be extended further. As such, any data presented does not include target speeds of 10 pixels/s unless specified otherwise.

### Computing the nonlinear inhibiting signal

The absolute local spatio-temporally averaged estimate of optic flow from the MLI was nonlinearly conditioned and fed into multiple locations of the model target detection pipeline (see Fig. 2). More specifically, a Naka-Rushton (Naka and Rushton 1966) saturating nonlinearity was used to condition the inhibiting signal ($E_{\textrm{MLI}}$). The conditioned signal ($E_{\textrm{MLIci}}$) was calculated on a per-pixel (*i*) basis using Eq. 1:

1 $$\begin{aligned}{} & {} E_{\textrm{MLIci}} = \min \left( G\frac{E_{\textrm{MLIi}}}{E_{\textrm{MLIi}} + c}, 1\right) \end{aligned}$$

2 $$\begin{aligned}{} & {} y_{i} = x_i(1-E_{\textrm{MLIci}}) = {\max \left( \frac{x_ic - x_i(G-1)E_{\textrm{MLIi}}}{E_{\textrm{MLIi}} + c}, 0\right) }\nonumber \\ \end{aligned}$$

Here, the initial constant (*c*) was chosen to be the mean of the local motion output (0.05) at a background and target speed of 29 pixels/s as it was expected this would provide an initial operating point for all speeds (faster and slower). A gain (*G*) was used to observe the effects of under- and over-saturating the top end roll-off of the saturating nonlinearity. The conditioned inhibiting signal was subtracted from unity in order to produce a signal that approached 1 when there was no recorded local motion and approached 0 when there was a large amount of local motion, and hence higher probability of false detections. This inhibition map was then multiplied with the signal at the corresponding inhibition location (*x*) to give the inhibited output (*y*) (see Eq. 2).

### Determining inhibition locations

To find the best location for inhibition within the BIV model, four locations were examined (see Fig. 2). These four locations were selected as they were each separated by a nonlinear operation, meaning they were all distinct mathematically. Inhibition at **A** was at the input to the RTC, before the first nonlinearity, and had the ability to suppress information before it was rectified, split into positive and negative branches, and thresholds used to make decisions about possible target signals. This is because the early stages of the model enhance information that could be useful for multiple purposes, while latter stages, which are more specialised, remove information not necessary for specific purposes. Therefore, inhibition at this location allowed more flexibility as all the information still existed. The inhibition also only had to be applied to a single channel as the RTC separates falling and rising signals into two channels that flow through to the end of the model. Therefore, inhibition at this point is more computationally efficient.

Location **B** put the inhibition into the variable low-pass filters (VLPF’s) of the RTC to adjust the derivative threshold based on the local clutter level but before rectification at the end of the RTC. Since location **B** served as an adjustment to the thresholding operation, it was used in conjunction with inhibition at location **A**. As the inhibition at location **A** could suppress the signals, the change over time from a target may no longer be large enough to trigger the fast adaptation for rising signals due to the static threshold. To adapt to this, adjusting the threshold using inhibition at location **B** allowed those smaller changes to produce the correct rising responses through the RTC.

Location **C** placed the inhibition after the output of the RTC and at the input to the ESTMD. This suppressed the two rectified channels, differing from location **A** as two impulses that follow closely would interact at full strength, suppressing any secondary impulse. Inhibition at **A** had the ability to suppress the initial impulse before it interacted with the secondary impulse, reducing the amount of suppression on the secondary impulse which may have been a target. At location **C** the inhibition also has to be applied to the two rectified channels separately, potentially reducing efficiency.

Location **D** applied the inhibition to the output of the BIV model following the correlation of the two processing branches, effectively a post-processing method, i.e. it acted as a variable local threshold for determining what is a possible target and what is not. Inhibition at location **D** was equivalent to that used in (Wiederman et al. 2008b).

### Dynamic signal conditioning

To add another level of control to the lateral inhibition, signal analysis was performed (as shown in Eqs. 1 and 2) to find a link between the global motion and the value of the saturating nonlinearity, *c*. As previously stated, insects used the LPTCs to calculate global motion. However, the implementation of this cell, as outlined in (Brinkworth and O’Carroll 2009; Wiederman et al. 2008b), is outside the scope of this study. Instead, we used a simplification of the LPTC that relies on the mean of the absolute local area motion over the entire frame ($\overline{E_{\textrm{mli}}}$) to get an estimate of global motion.

Data from the global motion estimate and model performance for different values of *c* were collected to find a correlation between the two. Data was collected over the 6 training backgrounds (see Fig. 3a–f) to give a range of responses. Both the background and target speeds were matched using the 4 speeds mentioned previously in Sect. 2.2 giving a total of 24 scenarios. This avoided the difficult task of predicting target speed prior to observing it. The best performing value of *c* was taken from each simulation and used to calculate a function of best fit.

### Tuning the MLI temporal filters

Temporal filtering within the MLI, modelled using a first order low-pass filter, is necessary to reduce fluctuations and provide a smooth motion estimate. However, previous studies (Melville-Smith et al. 2019) suggest that the temporal low-pass filtering in the MLI can cause unwanted side-effects in the estimation of local area motion for the purpose of inhibition for target detection. These side effects include leading edges insufficiently suppressed, and suppression from trailing edges extending too long. To help eliminate these characteristics we tested corner frequency values from 0.453 to 6.0 Hz over the training set of backgrounds and all speeds to find a better operating point. Increasing the corner frequency (reducing the time constant) minimises temporal blurring and delay, which enhances the inhibition signal for the purposes of target detection at the cost of having a more temporally variable signal.

### Comparative methodology

To compare the performance of the proposed BIV ’22 model, the existing BIV ’08 and BIV ’19 algorithms were used. Previous research has shown that under similar testing methodologies to those used in this study, LCM performs better than STLC and RLCM (Melville-Smith 2021). For this reason we chose only to use LCM and FKRW for comparison. PBAS was considered; however, due to its poor performance in moving frames of reference and binary output, which limits the ability to use inhibition, results are not included here.

For model initialisation, 300 frames were used to allow the BIV model’s filters to stabilise, with the following 100 frames then used to compare performance. For FKRW and LCM only the last 100 frames were used as the models do not require parameter stabilisation since they have no temporal filtering components. For the FKRW method, as it was designed to look for brighter targets, the input frames were normalised between 0 and 1, then inverted (1-pixel intensity). This made the dark targets bright and allowed the algorithm to be used without further modification. This sequence was repeated for all 20 backgrounds, 4 target speeds and 4 background speeds, for a total of 320 scenarios for each algorithm. To observe the effects of inhibition on FKRW and LCM, the statically conditioned inhibition signal was obtained from the MLI, subtracted from unity, and then multiplied with the method’s raw result on a frame-wise basis.

Strictly speaking, all algorithms used really performed target enhancement, not target detection. A subsequent thresholding operation was required to take the saliency maps produced by the algorithms and determine what components would be classified as targets. In order to perform this thresholding operation a winner takes all algorithm was used with a 7$\times$7 kernel. This reduced the local clutter, leaving only the local maximum for FKRW and BIV, and local minimum for LCM (due to a negative local contrast on dark targets) to be found. For FKRW and LCM, if targets were detected within a 5$\times$5 kernel centred on the original target position, then it was declared a true detection, otherwise it was declared a false detection. For the BIV models, as this method has a temporal component and relies on the detection of the trailing edge of a moving target, detections were considered a true detection if they occurred within a 5$\times$5 kernel with its centre shifted 1 pixel to the right of the original target position. As the direction of travel of all targets is right-to-left, this single pixel shift would align the centre of the kernel with the trailing edge of any target.

To measure and compare the target detection performance of the algorithms, the AUROC curve was used (Hanley and McNeil 1982; Brown and Davis 2006). The value of the AUROCs was found by integrating the respective receiver operating characteristic (ROC) curves between FP values of 0 and 20, FP = 20 being chosen as a reasonable upper limit for a real-world application.

## Results

### Detection performance versus clutter

**Fig. 4:**
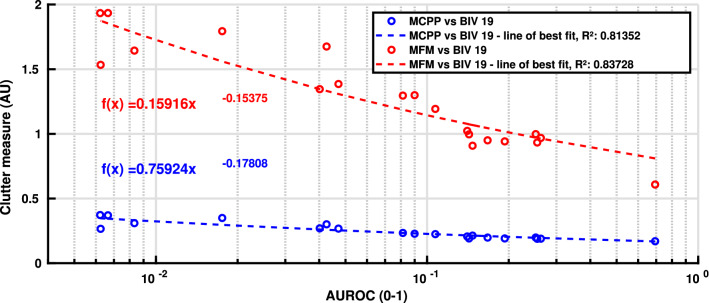
The BIV ’08 model’s (Wiederman et al. 2008a) median AUROC performance for all backgrounds and speeds versus two measures of clutter. This shows the negative correlation between the presence of clutter and performance of the BIV ’08 model. Clutter measures include the mean contrast per pixel (MCPP) (Skelton et al. 2019) and the mean frequency magnitude (MFM), which uses a Fast Fourier transform to compute the mean magnitude of high frequencies from 1.044 to 1.389 cycles/degree (2–2.7 pixels/cycle). The lines of best fit are power functions with parameters provided on the graph

The relationship between high-frequency spatial clutter and the BIV 08’s median area under the receiver operating characteristic (AUROC) performance for each background can be seen in Fig. 4. This figure shows a high (negative) correlation between the clutter measures and the performance of small target detection using the BIV ’08 model. The methods used for calculating the spatial clutter were the mean contrast per pixel method, as described in (Skelton et al. 2019) and the mean frequency magnitude for higher frequencies obtained from a 2D fast Fourier transform. The observed relationship between increasing clutter and decreasing performance (increasing false positive rates) is why it is believed that using local clutter estimations to alter the target detection processing would result in increased target detection rates.

### Model tuning

**Fig. 5:**
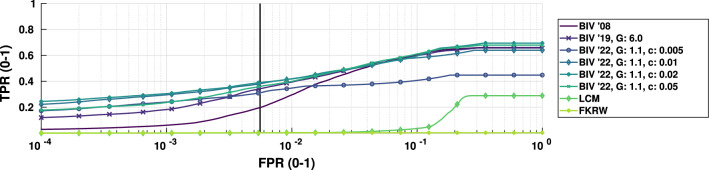
Performance of BIV ’22 compared to BIV ’19 (Melville-Smith et al. 2019), and BIV ’08 (Wiederman et al. 2008a) on the background ‘Lab’ with background and target speeds of 29 pixels/s. A false positive rate (FPR) of 1.0 represents 3582 false positives. The vertical black line represents 20 false positives. All nonlinear inhibition conditions show benefits for higher true positive rates (TPR) at lower FPR compared to other methods. Unlike linear signal conditioning, nonlinear conditioning provided better target separation at low FPRs, with the inhibitory drive more readily saturating with increasing levels of false positives. The relative performance of the comparison method, local contrast metric (LCM) was so low in this test that it did not detect any of the true targets until the false positive rate was almost 10x larger than the upper false positive threshold

**Fig. 6:**
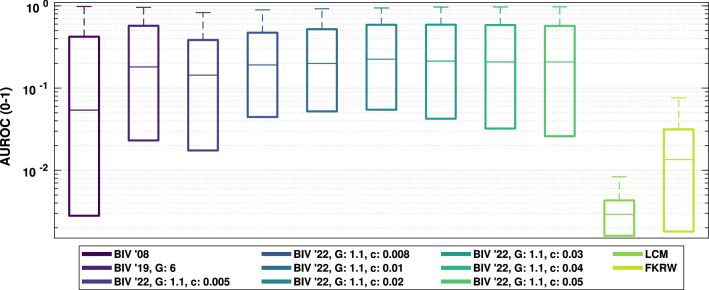
Performance of BIV ’22, up to 20 false positives, over all training backgrounds and speeds. Values of *G* = 1.1 and *c* = 0.02 gave the highest median performance. A linear gain of 6 was found to perform best for BIV ’19 (Melville-Smith et al. 2019) and is shown for comparison, as is the facet kernel random walker method (Qin et al. 2019). Other values of *G* are not shown for visual clarity. Note the logarithmic y-axis and the fact that the comparison method, facet kernel random walker (FKRW), performed well over 10x worse than the BIV ’22 algorithms

Initial results from the tuning data showed positive increases in performance. Figure 5 shows the results from the background ‘Lab’ with target and background speeds set to 29 pixels/s. Only results for $G = 1.1$ are shown for visual clarity and because this was the best performing value. The figure shows that all nonlinear settings present outperform both BIV ’08 and BIV ’19 (up to 20 FPs). Particular attention should be given to the early separation gains when fewer false positives occur. For general performance, the training set showed the best median operating point was with $G = 1.1$, and $c = 0.02$, for a maximum of FP = 20 (see Fig. 6).

Separating the scenes with manufactured structures from those without gave distinct operating points for best performance. Scenes with manufactured structures received the best AUROC score with $G = 1.1$ and $c = 0.008$. This low value of *c* was expected as indoor scenes typically have sharp (high intensity, high frequency) edges that come from manufactured objects such as walls and windows, while much of the environment is uniform and flat, resulting in lower local optic flow signals overall. Thus, to make the most of the available dynamic range, a steeper slope on the conditioning Naka-Rushton is required. Scenes without man-made objects performed best when $G = 1.1$ and $c = 0.04$. This is because the outdoor environments have more consistency in their high frequency elements across the images. This causes the local motion estimates to be much higher than those of indoor scenes and requires a flatter slope to make use of the dynamic range of the inhibiting signal if clipping is to be avoided. The common gain of 1.1 suggests that the introduction of clipping at the high end is beneficial, as it entirely removes FPs in the most cluttered areas of the scene due to the presence of hard saturation. From hereon in any mention of BIV ’22 will refer to a value of $G = 1.1$ and $c = 0.02$, unless specified otherwise.

### Target and background separation

**Fig. 7:**
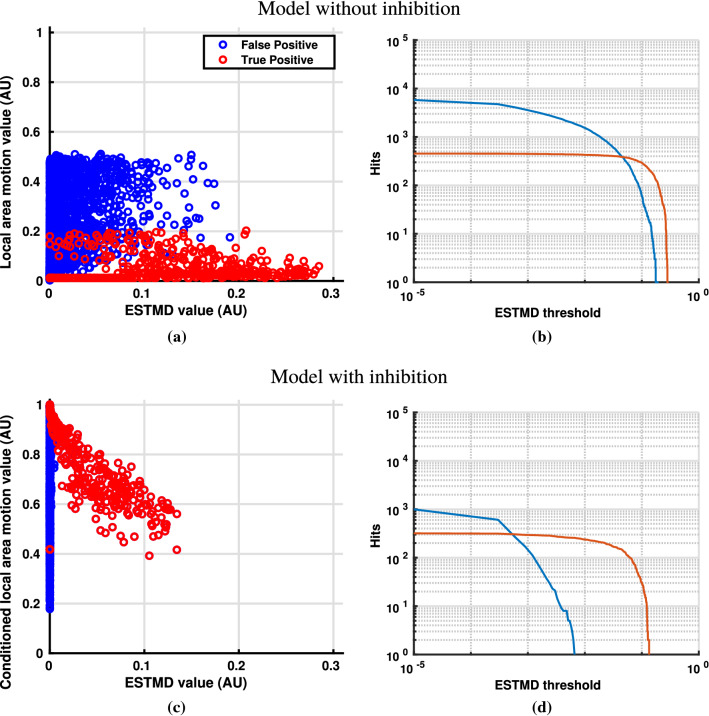
Detection of targets against the background ‘Rubble’ with a target and background speed of 29 pixels/s. **a** Shows BIV ’08’s ESTMD output (X-axis) and the raw MLI local area motion values (no nonlinear conditioning) used as a second dimension (Y-axis) for added separability. **b** Shows the cumulative distribution function of **a** using threshold values (X-axis) to binarise the output of the ESTMD and calculate detections. **c** Shows the ESTMD output of BIV ’22 with inhibition at point **A** (X-axis) and the nonlinearly conditioned local motion feedback values from the MLI. **d** Shows the cumulative distribution function of **c** (nonlinear inhibition model) using threshold values (X-axis) to binarise the output of the ESTMD and calculate detections.. The total number of false positives in **b**, **d** differ due to values in **d** being suppressed below the minimum value on the graph. These figures show that the use of inhibition has the ability to significantly increase target-background discrimination as the ESTMD threshold separation between the false and true targets (difference between the red and blue lines on the right graphs) is much greater in **d** than for **b**

Without local optic flow estimations, the algorithms were only able to separate targets from the background in a single dimension, using a threshold on the salience maps. Introducing an inhibiting signal based on local area motion allowed for a second dimension to help separate targets from the background. Figure  7a shows the output of BIV ’08’s ESTMD without any inhibition versus the original unconditioned local area motion calculated on the pixel on which the targets or background were detected.

Traditionally, all background and target detections would exist on the x-axis, the cumulative distribution function (CDF) for which can be seen in Fig. 7b. This shows that without utilising the optic flow signal it is possible to detect 100–130 targets before any false alarms. Introducing the local area motion estimation as a second dimension helps to improve discrimination between the background and targets. Figure 7c shows the results of conditioning the local area motion with the nonlinear transform and using it as an inhibiting signal at the start of the RTC (location **A** in Fig. 2). Often this led to very clear separation between the majority of targets and the background, with false positive intensity reduced and target intensity (true positives) largely unaffected or, if diminished, much less-so than the false alarm rates. The CDF with nonlinear inhibition applied at location **A** can be seen in Fig. 7d, where significant suppression of the background has increased separation allowing over 250 true positive (TP) detections before any false positives (FP) occur.

### Inhibition locations

**Fig. 8:**
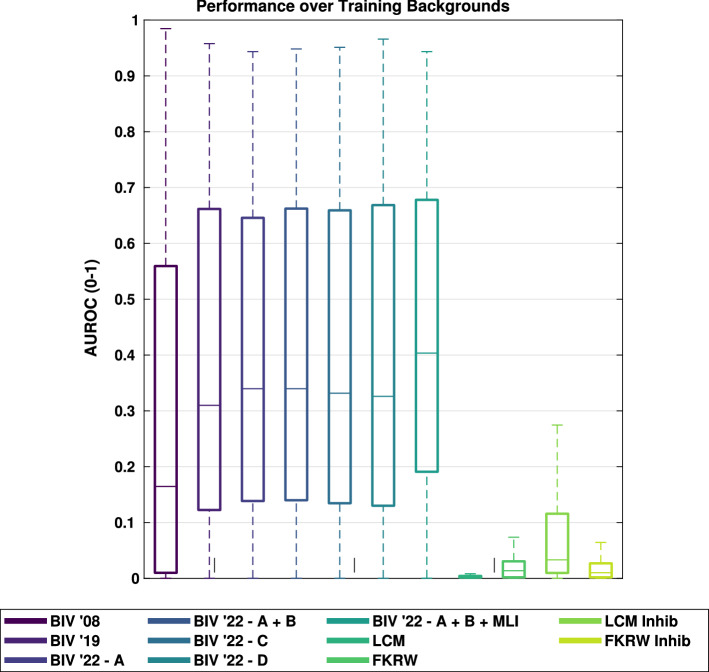
Comparative performance of BIV ’08, BIV ’19, and BIV ’22 with inhibition at different locations (**A–D**) and with a tuned temporal filter in the MLI. The results are shown for the training backgrounds over all speeds. LCM and FKRW performance are shown for comparison with and without inhibition. The 5th, 25th, median, 75th, and 95th percentiles are shown. Inhibition at location **A** and **B** performed better than other locations. Tuning the temporal filter in the MLI (corner frequency = 4 Hz) further improved performance. AUROC calculated by integrating up to 20 false positives. LCM and FKRW performed significantly worse than any of the BIV models

An examination of the pooled performance based on using the different inhibition locations and the entire training set of backgrounds showed that the best median performance was obtained by applying inhibition at both locations **A** and **B** together (see Fig. 8). Median performance for this combination was 2.8%, and 3.5% better than the individual locations **C**, and **D**, respectively. Compared to location **A** alone, inhibition at location **A** and **B** together had a minor increase in median performance; however, **A** and **B** together had larger 25th and 75th percentile values. This suggests it can be beneficial to use inhibition on the VLPF in the RTC (Location **B**) to adjust the threshold which determines whether a temporal change over a pixel requires a fast or slow adaptation state whenever inhibition occurs earlier in the model. Without inhibition at locations **A** and **B**, some target signals were suppressed to the point where they were smaller than the required threshold for a fast adaptation state within the VLPF and no longer produced the transients required for small target detection.

Using BIV ’22 with inhibition at location **A** and **B** as a benchmark and comparing results for each condition, paired t tests were performed on the results (see Table 1). The performance of BIV ’22 with inhibition at location **A** and **B** was significantly larger than all other methods (excluding MLI tuned methods) except for BIV ’22 with inhibition at location **D**, where no significant difference was measured. However, the p value was only non-significant following a Bonferroni post hoc correction indicating that there may be a slight difference between the two. The mean difference between the two datasets on a per-simulation basis, suggests that the performance of BIV ’22 with inhibition at **A** and **B** is generally larger than BIV ’22 with inhibition at **D** but further investigation is required to determine if this difference has any practical relevance.

**Table 1:** 25th, 50th and 75th AUROC percentiles for the training backgrounds over all speeds as shown in Fig. 8

Method	25th	Median	75th	Mean diff.	Paired t test	
					Stat. diff.	*p*
BIV ’08	0.0082	0.1710	0.5608	$-$ 0.1100	Yes	< 0.005
BIV ’19	0.1251	0.3083	0.6545	$-$ 0.0194	Yes	< 0.005
BIV ’22 (A)	0.1388	0.3363	0.6479	$-$ 0.0060	Yes	< 0.005
BIV ’22 (A + B)	0.1429	0.3378	0.6652	–	–	–
BIV ’22 (C)	0.1349	0.3284	0.6666	$-$ 0.0060	Yes	< 0.005
BIV ’22 (D)	0.1319	0.3260	0.6631	$-$ 0.0045	No	0.178
BIV ’22 (A + B + MLI)	0.1938	0.4016	0.6804	0.0303	Yes	< 0.005
LCM	0.0025	0.0030	0.0038	$-$ 0.4041	Yes	< 0.005
LCM Inhib	0.0101	0.0309	0.1150	$-$ 0.3363	Yes	< 0.005
FKRW	0.0018	0.0133	0.0262	$-$ 0.3869	Yes	< 0.005
FKRW Inhib	0.0015	0.0090	0.0204	$-$ 0.3877	Yes	< 0.005

The mean difference between the method and BIV ’22 with inhibition at location **A + B** (method—BIV ’22 (A + B)) is shown, with the difference being calculated on matched simulations. Paired T test results are also shown for each method compared to BIV ’22 (A + B), with a 95% confidence after a Bonferroni correction for the 10 tests

### Dynamic signal conditioning

**Fig. 9:**
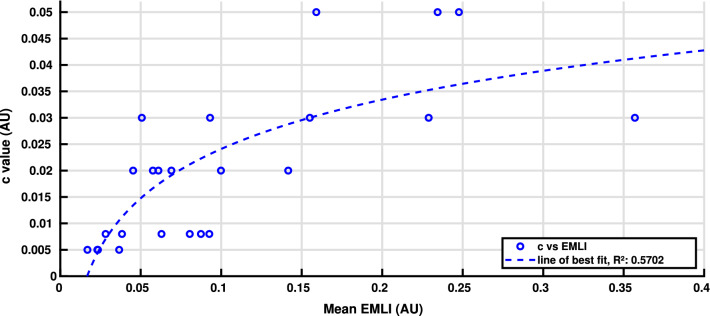
The correlation between best performing value of *c* for a given speed and background and mean of the local area motion over the entire frame ($G = 1.1$). Equation 3, the line of best fit, is also shown as this was used for dynamically adjusting the inhibiting signal

From individual training background results, a trend between background speed and the value of *c* was observed. Figure 9 shows the correlation between $\overline{E_{\textrm{mli}}}$ and the best performing *c* value with a *G* value of 1.1. The line of best fit is also shown and resulted in Eq. 3, which allows for a dynamic computation of *c* based on an estimate of global motion.

3 $$\begin{aligned} c = 0.013488 \times \textrm{ln}(\overline{E_{\textrm{MLI}}}) + 0.05514 \end{aligned}$$

### Temporal filtering in the MLI

**Fig. 10:**
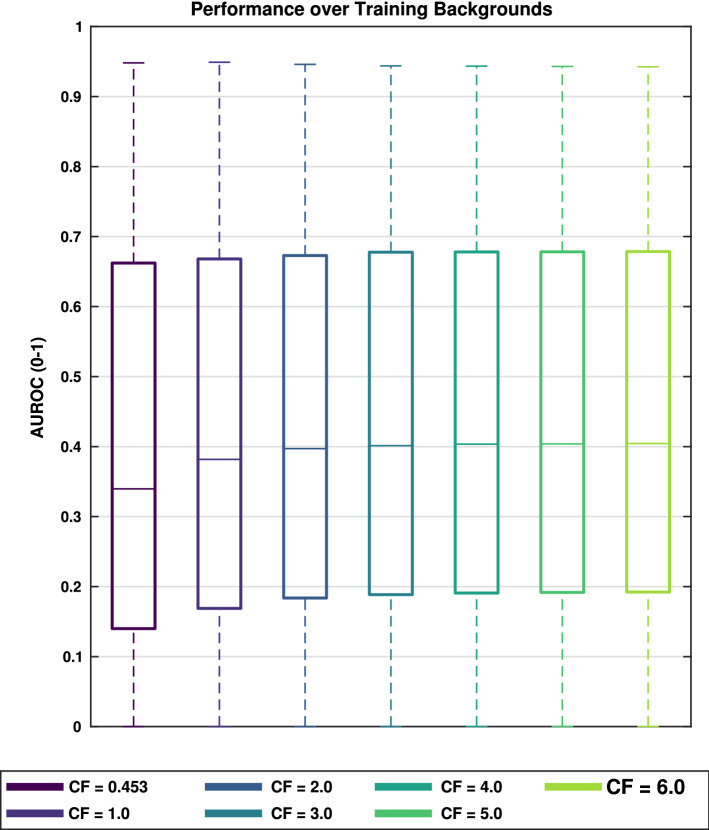
Performance comparison of different MLI temporal corner frequency (CF) values. Performance improvements start to drop off between a corner frequency of 3.0 and 4.0 for BIV ’22 with inhibition at location **A + B**. No meaningful performance difference was observed between models with the highest CF values used

**Fig. 11:**
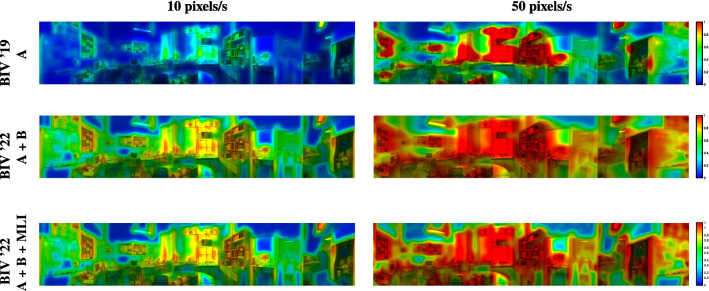
Inhibition maps for BIV ’19 (top) and BIV ’22 with the MLI temporal corner frequency set to 0.453 Hz (middle) and with a tuned temporal filter set to 4.0 Hz (bottom). Areas of dark red represent the highest levels of inhibition, yellow modest levels, and blue the lowest. The left and right columns show the responses for a background speed of 10 and 50 pixels/s, respectively. Nonlinear inhibition increases the overall amount of suppression (particularly in areas of mid-level optic flow), reduces the amount of saturation, and produces smoother roll-off between areas of differing clutter. The MLI tuning results in inhibition maps that are more condensed around the artefacts of clutter in the images at the leading edges (relative to the direction of camera rotation) while simultaneously expanding the amount of inhibition around the trailing edges of clutter objects. This compresses the red-to-yellow transitions towards the right hand side of clutter objects (the direction of camera rotation is left-to-right) and stretches them on the left hand side

Over the training backgrounds, tuning the MLI corner frequency showed large performance improvements over the original published value (0.453 Hz). Performance improvements began to taper off with a corner frequency value between 3.0 and 4.0 Hz, to a 18% and 19% performance increase from the original value, respectively. Figure 10 shows the performance of different corner frequency values. While this plateau in performance suggests that the temporal low-pass filter could be removed from the MLI, it would result in reduced accuracy of optic-flow calculations (Skelton et al. 2020). Having no low-pass filtering would cause larger fluctuation in the feedback, possibly leading to instability. Additionally, if the optic-flow stages were utilised, a trade-off may need to be made between target saliency improvement and motion vector accuracy by means of the temporal low-pass filters corner frequency. Due to these factors, we have decided to keep the low-pass filter intact. Figure 11 (bottom) shows the conditioned nonlinear inhibition map with an MLI corner frequency of 4 Hz. It can be seen that trailing edges of objects (right hand side) have been reduced and leading edges of objects increased compared to the conditioned nonlinear inhibition map with an MLI corner frequency of 0.453 Hz (middle). Henceforth, any mention of the tuned MLI will correspond to an MLI with a corner frequency of 4.0 Hz.

### Overall performance

**Fig. 12:**
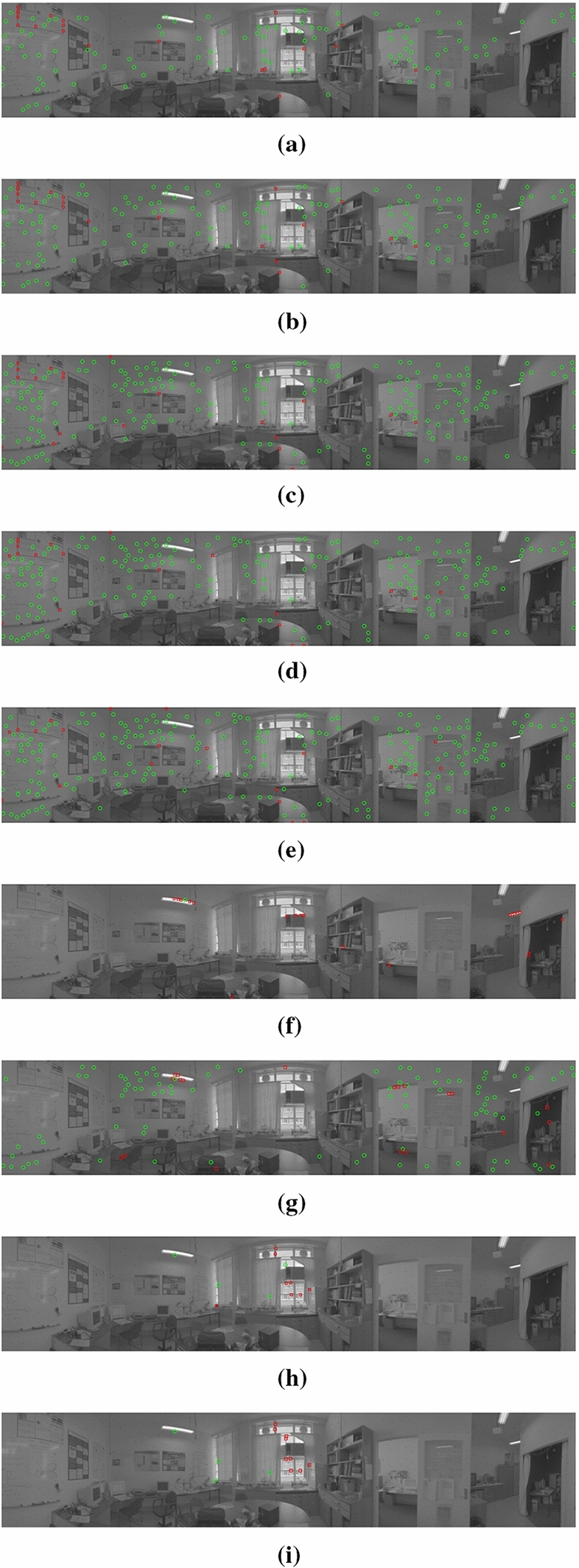
‘Lab’ input imagery with detection overlays for a fixed false positive rate of 0.0056 (20 FP). Green circles show correctly detected targets and red squares show false positives. Both the background and target speeds were set to 17 pixels/s. Images from top to bottom represent: **a** BIV ’08 with 108 TP; **b** BIV ’19 with 142 TP; **c** BIV ’22 with inhibition at location **A** with 187 TP; **d** BIV ’22 with inhibition at location **A** and **B** with 189 TP; **e** BIV ’22 with inhibition at location **A** and **B**, and tuned MLI, with 218 TP; **f** LCM, with 1 TP; **g** LCM with inhibition, with 90 TP; **h** FKRW, with 4 TP; **i** FKRW with inhibition, with 4 TP. FKRW was unable to find more than 13 targets in this example

The introduction of nonlinear inhibition showed great improvement in detection performance, especially for backgrounds containing physically large man-made features. Figure 12 shows the detection performance for up to 20 FPs for a single scene, target and background speed combination for BIV ’08 Fig. 12a, BIV ’19 Fig. 12b and BIV ’22 with inhibition at location **A** Fig. 12c. Both LCM and FKRW with and without nonlinear inhibition are also shown.

BIV ’08 obtained 108 TPs, while BIV ’19 obtained 142 TPs: a 31% improvement. BIV ’22 with inhibition at location **A** further improved performance, obtaining 187 TPs: a 73% increase over BIV ’08 and 32% increase over BIV ’19; and an insight into the effects using linear versus nonlinear conditioning.

Figure 12d shows BIV ’22 (without the tuned MLI temporal filter) with inhibition at locations **A** and **B** obtaining 189 TPs. The inclusion of the tuned MLI temporal filter (corner frequency of 4.0 Hz) further improved the number of detections to 218 TPs: an 15% increase over BIV ’22 without MLI tuning, and a 102% and 53% increase over BIV ’08 and BIV ’19, respectively.

Figure 12f, g shows LCM without and with inhibition, respectively. LCM without inhibition obtains 1 TP, while the use of inhibition increases the TPs to 90, a 900% increase. Figure 12h, i shows FKRW without and with inhibition, respectively. Both methods obtained 4 TPs suggesting no advantage from inhibition at the location it was applied. FKRW was only able to find 13 potential targets in this example with 9 of them being FPs.

Comparing the maps of linear and nonlinear inhibition (top vs. middle and lower images of Fig. 11), it can be seen that nonlinear conditioning generates smoother falloff between different levels of suppression than the linear conditioning, which produces three distinct levels with little in-between, i.e. the colour changes for the nonlinear inhibition are more gradual. Also, nonlinear inhibition does not saturate (completely suppress) large areas of image in the way that linear inhibition does: more of the signals remain intact, albeit reduced in amplitude. Consequently, more targets can be detected because the falloff produces a more gracefully decaying distribution of inhibition.

**Fig. 13:**
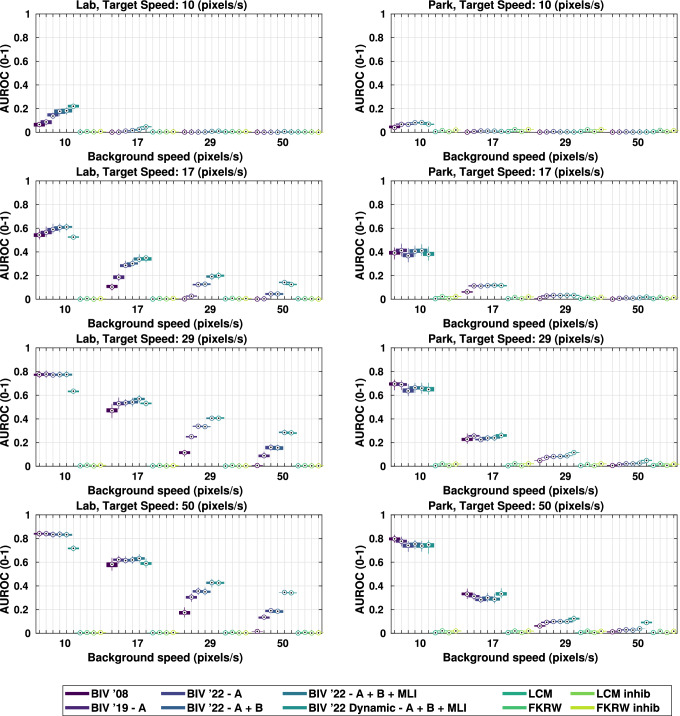
Box and whisker plots (5th, 25th, median, 75th, 95th) for the AUROC performance for up to 20 false positives for all speed scenarios on the backgrounds ‘Lab’ (man-made elements) and ‘Park’ (natural elements). Target detection in natural scenes is often more difficult due to the continuous spatial clutter versus the high intensity local spatial clutter of a scene with large man-made objects in it. FKRW and LCM detected very few true targets and performed worse than all BIV model configurations under all conditions except for target speeds of 10 pixels/s, which were below the detection tuning for the BIV models

Figure 13 shows the variability in performance for the backgrounds ‘Lab’ (an example of a man-made scene) and ‘Park’ (an example of a natural scene) under all target/background speed conditions. The faster the targets moved relative to their backgrounds the easier they were to detect. For ‘Lab’, BIV ’22 always performed better or as well as BIV ’08 and BIV ’19. Similarly, for ‘Park’, BIV ’22 methods performed better or as well as BIV ’08 and BIV ’19, as long as the target moved no faster than the background. The exception to this was BIV ’22 with dynamic inhibition which outperformed or was equal to BIV ’08 and BIV ’19 under all but two speed combinations.

BIV ’22 with dynamic inhibition performed better as well as BIV ’22 with static inhibition on ‘Lab’, as long as the targets moved slower or at the same speed as the background. This is because the dynamic feedback was derived from the mean local area motion of the frame, upon which the target motion has an almost negligible impact, i.e. there is no knowledge of target speed. On ‘Park’ BIV ’22 with dynamic inhibition outperformed linear feedback more frequently than BIV ’22 with static inhibition.

Another point of interest is that the dynamic inhibition performance is much better than that of the static inhibition for Park compared to Lab. This is thought to be due to the large amount of clutter in Park compared to Lab, as this allows a more accurate estimate of motion to be obtained and thus a better estimate of *c*.

Performance of BIV ’08 was larger than FKRW, and LCM under most conditions (see Tables 2 and 3), and for almost all speed conditions (Table 2), BIV ’22 with either static or dynamic inhibition generally outperformed BIV ’08. For motion that fell outside of the operational range of the model (a target speed of 10 pixels/s), the use of inhibition increased performance significantly. However, within this region FKRW was competitive often matching or outperforming the BIV methods. This highlights the BIV’s reliance on the temporal component of targets in imagery and the importance of correctly tuned filters for different speed settings. At these levels of motion a smaller value of *c* in the feedback (a steeper remapping gradient) can be beneficial as the background signals dominate the target signals. As a result, an increase in suppression is more likely to reduce FPs than TPs. However, in a real-world application it may be difficult to know what the target speed is ahead of time, making it hard to optimise such criteria. When targets are moving much faster than the background, such as targets at 50 pixels/s and background at 10 pixels/s, BIV ’08 performs best. This is because the temporal energy of the targets alone is sufficient to separate them from the background. Under this condition, BIV ’22 with dynamic inhibition performed the worst out of all BIV methods due to its reliance on background velocity estimation.

**Table 2:** 25th, 50th and 75th AUROC percentiles for all methods on individual target (T) and background (BG) speed combinations, over all backgrounds (up to 20 false positives)

Speed	BIV ’08			BIV ’19			BIV ’22—A + B + MLI			BIV ’22 Dynamic			LCM			FKRW		
	(Wiederman et al. 2008a)			(Melville-Smith et al. 2019)									(Chen et al. 2014)			(Qin et al. 2019)		
[T, BG]	25th	Med	75th	25th	Med	75th	25th	Med	75th	25th	Med	75th	25th	Med	75th	25th	Med	75th
[10, 10]	0.028	0.083	0.175	0.063	0.135	0.266	**0.085**	**0.194**	**0.341**	0.068	0.159	0.272	0.002	0.004	0.006	0.002	0.054	0.106
[10, 17]	0.000	0.001	0.003	0.003	0.011	0.028	0.015	0.044	0.125	0.014	0.049	0.116	0.002	0.003	0.006	**0.003**	**0.050**	**0.100**
[10, 29]	0.000	0.001	0.002	0.001	0.003	0.006	0.004	0.018	0.079	0.003	0.011	0.057	0.002	0.004	0.006	**0.004**	**0.041**	**0.101**
[10, 50]	0.000	0.001	0.002	0.001	0.002	0.005	0.003	0.015	0.088	0.002	0.006	0.062	0.002	0.004	0.006	**0.003**	**0.036**	**0.104**
[17, 10]	0.397	0.563	0.789	0.435	0.599	0.814	**0.438**	**0.640**	**0.810**	0.420	0.530	0.696	0.002	0.004	0.006	0.003	0.048	0.106
[17, 17]	0.038	0.117	0.251	0.114	0.198	0.402	0.133	0.306	0.479	**0.135**	**0.341**	**0.473**	0.002	0.004	0.006	0.002	0.053	0.105
[17, 29]	0.002	0.006	0.018	0.035	0.063	0.200	**0.062**	**0.171**	**0.340**	0.059	0.167	0.321	0.002	0.004	0.006	0.004	0.051	0.100
[17, 50]	0.001	0.002	0.003	0.010	0.033	0.099	**0.041**	**0.113**	**0.259**	0.035	0.108	0.238	0.002	0.004	0.006	0.003	0.049	0.102
[29, 10]	0.676	0.811	0.957	**0.682**	**0.813**	**0.934**	0.664	0.806	0.914	0.631	0.735	0.853	0.002	0.004	0.006	0.004	0.038	0.102
[29, 17]	0.224	0.445	0.704	0.303	0.504	0.678	**0.281**	**0.542**	**0.652**	0.313	0.536	0.655	0.002	0.004	0.006	0.003	0.041	0.106
[29, 29]	0.035	0.117	0.236	0.122	0.238	0.426	0.128	0.324	0.470	**0.144**	**0.370**	**0.483**	0.002	0.004	0.006	0.002	0.054	0.106
[29, 50]	0.007	0.020	0.078	0.059	0.099	0.243	0.076	0.203	0.363	**0.088**	**0.246**	**0.383**	0.002	0.004	0.007	0.004	0.037	0.101
[50, 10]	**0.778**	**0.873**	**0.973**	0.766	0.860	0.945	0.733	0.848	0.925	0.710	0.786	0.880	0.002	0.004	0.007	0.004	0.036	0.106
[50, 17]	0.332	0.550	0.787	0.368	0.589	0.717	**0.332**	**0.601**	**0.698**	0.387	0.598	0.695	0.002	0.004	0.007	0.004	0.034	0.106
[50, 29]	0.052	0.169	0.323	0.137	0.281	0.454	0.133	0.323	0.512	**0.171**	**0.397**	**0.518**	0.002	0.004	0.007	0.004	0.042	0.104
[50, 50]	0.012	0.030	0.109	0.062	0.115	0.283	0.079	0.211	0.372	**0.105**	**0.275**	**0.416**	0.002	0.004	0.006	0.002	0.055	0.109

The best performing (median) method for each speed is highlighted in bold

**Table 3:** AUROC median performance, with 25th and 75th percentiles, for all methods on individual and grouped backgrounds, over all speed configurations (up to 20 false positives)

Background	BIV ’08			BIV ’19			BIV ’22—A + B + MLI			BIV ’22 Dynamic			LCM			FKRW		
	(Wiederman et al. 2008a)			(Melville-Smith et al. 2019)									(Chen et al. 2014)			(Qin et al. 2019)		
	25th	Med	75th	25th	Med	75th	25th	Med	75th	25th	Med	75th	25th	Med	75th	25th	Med	75th
All	0.017	0.178	0.668	0.106	0.333	0.668	0.158	0.400	0.671	**0.175**	**0.416**	**0.652**	0.002	0.004	0.006	0.003	0.040	0.105
Manufactured	0.015	0.217	0.730	0.218	0.442	0.736	**0.358**	**0.523**	**0.731**	0.371	0.515	0.682	0.002	0.004	0.007	0.004	0.031	0.098
Natural	0.019	0.166	0.580	0.079	0.269	0.600	0.091	0.266	0.597	**0.113**	**0.295**	**0.598**	0.002	0.004	0.006	0.002	0.055	0.111
Field (n)	**0.606**	**0.919**	**0.993**	0.642	0.873	0.991	0.674	0.851	0.985	0.647	0.805	0.934	0.006	0.009	0.011	0.332	0.343	0.359
Fountain (m)	0.092	0.363	0.824	0.265	0.435	0.773	0.374	0.495	0.767	**0.417**	**0.507**	**0.716**	0.004	0.006	0.008	0.182	0.194	0.203
Rubble (n)	0.222	0.442	0.841	0.483	0.577	0.765	0.489	0.577	0.764	**0.520**	**0.598**	**0.749**	0.003	0.004	0.006	0.111	0.120	0.128
Lounge (m)	0.201	0.425	0.754	0.562	0.702	0.814	**0.665**	**0.738**	**0.830**	0.636	0.703	0.742	0.002	0.003	0.004	0.018	0.019	0.023
Walkway (m)	0.128	0.350	0.804	0.380	0.538	0.812	0.432	0.552	0.794	**0.482**	**0.572**	**0.736**	0.002	0.003	0.005	0.087	0.094	0.101
Outdoor (n)	0.030	0.201	0.696	0.092	0.269	0.631	0.165	0.289	0.594	**0.215**	**0.309**	**0.600**	0.002	0.004	0.006	0.155	0.165	0.177
House (m)	0.010	0.214	0.834	0.106	0.293	0.758	0.209	0.373	0.729	**0.258**	**0.399**	**0.722**	0.009	0.011	0.015	0.078	0.085	0.095
Bluff (n)	0.113	0.225	0.778	0.150	0.313	0.750	0.211	0.343	0.716	**0.278**	**0.391**	**0.712**	0.001	0.003	0.004	0.080	0.085	0.090
Library (m)	0.051	0.196	0.702	0.276	0.446	0.659	0.368	0.477	0.653	**0.394**	**0.483**	**0.624**	0.005	0.007	0.009	0.085	0.096	0.105
Hill (n)	0.050	0.154	0.549	0.048	0.162	0.486	0.083	0.173	0.429	**0.126**	**0.213**	**0.467**	0.003	0.006	0.009	0.090	0.099	0.105
Lab (m)	0.012	0.138	0.558	0.123	0.268	0.599	0.310	0.415	0.620	**0.309**	**0.416**	**0.559**	0.001	0.002	0.004	0.004	0.007	0.009
Rock Garden (n)	0.069	0.119	0.503	0.092	0.161	0.464	0.112	0.172	0.454	**0.125**	**0.201**	**0.475**	0.002	0.003	0.005	0.090	0.097	0.104
Park (n)	0.008	0.062	0.362	0.024	0.104	0.361	0.037	0.112	0.354	**0.074**	**0.120**	**0.354**	0.003	0.005	0.006	0.016	0.018	0.021
Bushes (n)	0.020	0.066	0.380	0.064	0.115	0.422	0.075	0.100	0.374	**0.080**	**0.136**	**0.396**	0.002	0.003	0.005	0.000	0.002	0.003
Shadow (n)	0.021	0.061	0.390	0.059	0.134	0.382	0.079	0.138	0.355	**0.103**	**0.155**	**0.380**	0.003	0.004	0.006	0.026	0.030	0.036
Tree (n)	0.015	0.031	0.237	0.071	0.129	0.393	0.078	0.130	0.364	**0.095**	**0.154**	**0.385**	0.001	0.002	0.004	0.002	0.002	0.004
Car Park (m)	0.007	0.019	0.119	0.080	0.220	0.405	0.277	0.431	0.559	**0.310**	**0.442**	**0.576**	0.002	0.003	0.004	0.001	0.003	0.004
Botanic (n)	0.004	0.012	0.163	0.011	0.037	0.212	**0.019**	**0.043**	**0.207**	0.024	0.042	0.211	0.002	0.003	0.004	0.000	0.002	0.002
Creek Bed (n)	0.004	0.014	0.174	0.020	0.066	0.283	0.023	0.075	0.293	**0.026**	**0.083**	**0.283**	0.002	0.003	0.004	0.000	0.000	0.002
Classroom (m)	0.003	0.010	0.452	0.158	0.369	0.634	**0.336**	**0.487**	**0.692**	0.335	0.474	0.659	0.002	0.003	0.005	0.000	0.002	0.003

Backgrounds are labelled as natural (n) or manufactured (m) to indicate which group they belong to. The best performing (median) method for a particular background is highlighted in bold

**Table 4:** AUROC performance comparison for different methods

Method	Without targets at 10 pixels/s			With targets at 10 pixels/s		
	25th	Median	75th	25th	Median	75th
BIV ’08	1.000	1.000	1.000	1.000	1.000	1.000
BIV ’19	1.005	1.503	4.266	1.021	1.665	5.663
BIV ’22—A	0.982	1.484	4.788	1.003	1.943	10.411
BIV ’22—A + B	0.990	1.504	4.889	1.015	1.977	10.740
BIV ’22—C	0.989	1.481	4.614	1.010	1.948	10.000
BIV ’22—D	1.010	1.524	4.684	1.042	1.852	7.543
BIV ’22—A + B + MLI	0.995	1.688	5.764	1.046	2.243	14.731
BIV ’22—Dynamic	0.956	1.855	6.428	1.010	2.294	14.385
LCM	0.007	0.018	0.174	0.009	0.045	0.833
FKRW	0.041	0.217	0.792	0.079	0.368	2.864
LCM w/ Inhibition	0.063	0.327	1.722	0.107	0.686	12.881
FKRW w/ Inhibition	0.032	0.224	0.770	0.069	0.369	2.408

Performance is normalised against BIV ’08, with performance for 10 pixels/s targets shown as excluded and included

**Table 5:** 25th, 50th and 75th AUROC percentiles for the all backgrounds and speeds

Method	25th	Median	75th	Mean diff.	Paired t test	
					Stat. diff.	*p*
BIV ’08	0.0161	0.1747	0.6722	$-$ 0.0995	Yes	< 0.001
BIV ’19	0.1070	0.3276	0.6697	$-$ 0.0314	Yes	< 0.001
BIV ’22 (A)	0.1164	0.3398	0.6427	$-$ 0.0291	Yes	< 0.001
BIV ’22 (A + B)	0.1211	0.3451	0.6564	$-$ 0.0241	Yes	< 0.001
BIV ’22 (C)	0.1117	0.3417	0.6599	$-$ 0.0274	Yes	< 0.001
BIV ’22 (D)	0.1107	0.3391	0.6728	$-$ 0.0221	Yes	< 0.001
BIV ’22 (A + B + MLI)	0.1603	0.4001	0.6689	–	–	–
BIV ’22 Dynamic	0.1731	0.4155	0.6484	$-$ 0.0046	No	0.1883
LCM	0.0029	0.0039	0.0053	$-$ 0.4240	Yes	< 0.001
LCM Inhib	0.0140	0.0341	0.1328	$-$ 0.3491	Yes	< 0.001
FKRW	0.0028	0.0353	0.0861	$-$ 0.3672	Yes	< 0.001
FKRW Inhib	0.0023	0.0343	0.0984	$-$ 0.3627	Yes	< 0.001

The mean difference between the method and BIV ’22 with inhibition at location **A + B + MLI** (method—BIV ’22 (A + B + MLI)) is shown, with the difference being calculated on matched simulations. Paired T test results are also shown for the each method compared to BIV ’22 (A + B + MLI), with a 95% confidence after a Bonferroni correction for the 11 tests

For backgrounds that are minimally cluttered (e.g. Field), BIV ’08 provided the best detection rates. This is because the background signals interfere less with the target signals when compared to more cluttered scenes. In other words, any BIV ’22 inhibition introduced likely suppressed the targets more than the false positives. Nonlinear inhibition improved performance most on scenes containing man-made elements (Table 3), noting the median AUROC for nonlinear inhibition on man-made scenes is almost twice that for natural scenes. The likely reason for this is the additional high frequency information (texture) present in natural scenes.

**Table 6:** AUROC median performance, with 25th and 75th percentile, for FKRW and LCM with inhibition applied on individual backgrounds and grouped backgrounds, over all speed configurations, up to 20 false positives

Background	FKRW w inhibition			LCM w inhibition			FKRW improvement (%)			LCM improvement (%)		
	25th	Med	75th	25th	Med	75th	25th	Med	75th	25th	Med	75th
All backgrounds	0.003	0.039	0.110	0.013	0.036	0.139	$-$ 23.5	$-$ 0.8	4.9	495.5	830.8	2165.0
Manufactured backgrounds	0.004	0.024	0.102	0.039	0.114	0.184	$-$ 16.7	$-$ 23.3	4.4	1693.0	2690.9	2585.8
Natural backgrounds	0.002	0.047	0.115	0.009	0.019	0.049	5.0	$-$ 13.7	4.0	322.7	401.3	764.5
Field (n)	0.342	0.362	0.380	0.145	0.297	0.352	2.8	5.7	5.6	2276.2	3392.9	3101.1
Fountain (m)	0.168	0.196	0.216	0.130	0.156	0.172	$-$ 7.8	1.2	6.1	3032.5	2386.9	2063.5
Rubble (n)	0.214	0.237	0.252	0.166	0.282	0.346	92.3	96.9	96.7	5013.1	6449.4	5788.1
Lounge (m)	0.014	0.017	0.020	0.067	0.160	0.312	$-$ 20.6	$-$ 14.9	$-$ 14.0	3447.4	5245.0	7592.0
Walkway (m)	0.079	0.090	0.107	0.059	0.203	0.235	$-$ 9.4	$-$ 4.6	6.0	3351.5	6239.1	4910.1
Outdoor (n)	0.138	0.153	0.166	0.020	0.028	0.040	$-$ 11.1	$-$ 7.7	$-$ 5.9	807.9	624.7	539.7
House (m)	0.064	0.073	0.086	0.008	0.019	0.031	$-$ 17.3	$-$ 14.3	$-$ 10.3	$-$ 10.3	68.9	113.7
Bluff (n)	0.078	0.092	0.105	0.017	0.036	0.057	$-$ 2.9	8.3	16.3	1454.5	1210.9	1175.8
Library (m)	0.100	0.122	0.152	0.028	0.043	0.061	17.6	27.5	44.9	430.3	496.9	574.2
Hill (n)	0.073	0.090	0.100	0.014	0.016	0.020	$-$ 18.3	$-$ 9.0	$-$ 5.4	300.0	187.7	119.3
Lab (m)	0.002	0.004	0.007	0.048	0.102	0.145	$-$ 50.6	$-$ 39.4	$-$ 20.5	3325.0	5016.3	3774.7
Rock Garden (n)	0.089	0.096	0.104	0.029	0.058	0.077	$-$ 0.5	$-$ 0.9	0.5	1218.2	1669.7	1537.2
Park (n)	0.016	0.018	0.022	0.007	0.010	0.015	1.3	1.1	1.6	117.6	110.3	144.5
Bushes (n)	0.000	0.001	0.002	0.028	0.036	0.046	0.0	$-$ 26.7	$-$ 36.0	1246.3	1037.5	883.0
Shadow (n)	0.019	0.026	0.033	0.011	0.015	0.021	$-$ 28.1	$-$ 12.6	$-$ 8.3	231.3	254.1	286.4
Tree (n)	0.002	0.002	0.004	0.009	0.014	0.018	$-$ 15.8	10.0	8.1	615.4	537.2	365.8
Car Park (m)	0.000	0.002	0.003	0.167	0.210	0.230	$-$ 100.0	$-$ 24.0	$-$ 13.2	9200.0	6169.0	5248.8
Botanic (n)	0.000	0.001	0.002	0.004	0.006	0.008	0.0	$-$ 31.3	5.3	150.0	102.4	87.7
Creek Bed (m)	0.000	0.000	0.002	0.004	0.005	0.006	0.0	0.0	18.8	140.0	64.3	60.6
Classroom (m)	0.000	0.001	0.002	0.031	0.086	0.165	$-$ 100.0	$-$ 18.8	$-$ 31.0	1707.4	2750.0	3522.5
Average improvement							$-$ 13.4	$-$ 2.7	3.3	1902.7	2200.7	2094.4

Backgrounds are labelled as natural (n) or manufactured (m) to define which group they belong to. Performance improvement/deterioration of both FKRW and LCM with feedback are shown as a percentage of their respective method without feedback

In general, when not including target speeds of 10 pixels/s, BIV ’22 with dynamic inhibition performed the best. When aggregating results for all backgrounds (Tables 3 and 4) BIV ’22 had a median AUROC 2.33 times greater than BIV ’08, 1.25 times greater than BIV ’19, 10 times greater than FKRW, and 104 times greater than LCM. The inclusion of targets moving at 10 pixels/s reduces the median AUROC significantly. However, this increases the relative benefit from inhibition compared to the other BIV models. BIV ’22 with dynamic inhibition performed 3.97 times greater than BIV ’08, 1.44 times greater than BIV ’19, but reduces the benefits compared to other models, 7.4 times greater than FKRW, and 76.6 times greater than LCM. Overall, while motion of 10 pixels/s is outside the operating range of the BIV model, benefit can still be had using nonlinear inhibition. Furthermore, tuning the temporal and spatial filters to be sensitive to a different range of target speeds is expected to improve results for those speeds.

Overall, BIV ’22 with inhibition at location **A** and **B** with MLI tuning performs significantly better than all other methods except for BIV ’22 with dynamic inhibition, for which there is no significant difference in performance (see Table 5). This is likely due to BIV with dynamic inhibition having larger variation in performance due to its dependence on background speeds.

### Inhibition applied to other methods

The use of optic-flow for inhibition was also shown to be beneficial to the LCM technique, with average median performance on a scene increasing by 22 times (Table 6). This improved LCM performance above that of FKRW. LCM’s performance on a per frame basis increased from a median of 1.8% of BIV ’08 to 32.7% (Table 4).

FKRW did not show the same improvement, delivering similar performance both with and without inhibition. The absence of any improvement is thought due to the adaptive threshold within the FKRW algorithm, which is applied after the facet kernel. Many of the targets missed are within cluttered or darker areas, where contrast between the target and background is lower; and it is believed that the FKRW adaptive threshold is being set to capture higher contrast targets, such as dark targets against a bright sky. FKRW therefore completely removes lower contrast targets. As the optic-flow is most apparent in regions of clutter, the majority of suppression will occur where the adaptive threshold has already completely removed targets. This means that no further separation can be given to targets outside regions of clutter. As a result, current performance does not improve.

For this reason, in order for optic-flow inhibition to be useful, an algorithm must be able to compute some form of target probability (saliency). Unfortunately, FKRW does not retain such information. That said, it is believed that using optic-flow inhibition after the facet kernel, but before the adaptive threshold, could produce better results. LCM’s improved performance comes from every pixel having a probability associated with it based on its degree of contrast, which allows the inhibition to allocate higher probability to targets in areas of little clutter while reducing it for those in regions of high clutter.

## Discussion

Biological explorations have previously looked at global motion feedback within the vision of insects (Egelhaaf 1985; Warzecha et al. 1993); however no indication of local-area feedback has been noted. Wiederman, Brinkworth and O’Carroll (Wiederman et al. 2008b) took an engineering approach to suggest local motion inhibition within the BIV could offer advantages to real-world applications whilst still being biologically plausible. Our previous work (Melville-Smith et al. 2019) made modification to (Wiederman et al. 2008b) and showed the benefit of using linear local motion inhibition earlier in the BIV model. This aided the BIV’s pixel sized target detection performance, far exceeding the capability of any other state-of-the-art methods by reducing sensitivity to potential targets in areas of clutter, thereby reducing false positives in those areas.

In this study, applying a biologically plausible, dynamic, nonlinear inhibition mechanism further improved the small target detection capabilities of the BIV model relative to our earlier work. The current model has a general median AUROC 2.33 times that of BIV ’08 and 1.25 times that of BIV ’19. Performance over other methods were consistent with previous studies (Melville-Smith et al. 2019), with a median AUROC 10 times that of FKRW, and 104 times that of LCM.

For target speeds of 10 pixels/s, inhibition increased performance significantly. This was most useful when the target and background moved at the same speed, i.e. the targets were effectively static within their environment, the camera platform inducing all motion. This scenario represents those tested in (Wiederman et al. 2008a, b, 2010), which also showed a falloff in performance as target motion fell outside the speeds for which the models were tuned.

For target speeds of 10 pixels/s, when the backgrounds began to move faster than the targets, the performance of all BIV methods dropped below that of FKRW. This highlights the dependence of the BIV on temporal information. Spatial-only methods, such as FKRW, do not suffer accordingly. As a result, although in general its performance is lower than that of the BIV, FKRW performed more consistently over all speeds, while that of the BIV methods tend to fluctuate according to the target/background motion or scene properties. To get around the BIV’s performance drop off outside its tuned regime, multiple parallel models, each with differing tuning parameters, could be used and the outcomes fused. This would increase performance over a wider range of speeds.

The study shows that dynamic feedback can be beneficial, with performance dependent upon the accuracy of the global motion estimate. The method used to estimate global motion in this study was sufficient to show such merit. However, results suggest that the estimate of global motion was not terribly accurate for indoor scenes (see Lab and Lounge performance in Table 3), where there is often a lack of spatial features. It is believed that using the modified motion pipeline implementation from (Skelton et al. 2019) could provide more consistent motion estimates across all scenes and thus offer more uniform improvements independent of any scene characteristics. Other sensors, such as gyroscopes, could be utilised in a real-world setting to get additional information about the rotational velocity of the platform and help inform the algorithm.

Initial tuning of the inhibiting signal found that for more natural scenes a larger value of *c* (shallower slope) is preferred as this offers consistent global motion estimates over the entirety of a scene. This suggests that, not only are the target speeds an important factor in the performance of the BIV, but so too is the internal construction of a scene. Additional analysis is needed to determine what in a scene’s structure could be used to generate a more robust dynamic inhibition signal. It may also be possible to obtain further performance increases by modifying the spatial filter in the MLI. However, it is expected any such improvements would likely be minor compared to tuning the temporal filter as smaller kernels are expected to suppress targets more in areas of low clutter while larger kernels may not create a feedback map with sufficient detail to suppress smaller areas of clutter. As this study focused on analysing inhibition locations and the effects over entire scenes, further studies may also be warranted to look at the inhibition location performance based on localised areas of clutter. This could give a deeper understanding of what happens to the detection of both targets and clutter in these regions, leading to new inhibition schemes.

Inhibition on other algorithms (such as LCM) can be beneficial. However, this relies on it being applied before decisions are made on what is or is not a target. In other words, optic-flow inhibition could be applied to almost any algorithm to provide improved performance so long as the algorithm provides the probability of a target’s existence (salience maps), and not just a binary outcome regarding detection. Without this, inhibition will likely offer little or no benefit.

## Conclusion

This study investigated local motion feedback points within the BIV model. It showed that performance gains could be obtained by introducing inhibition concurrently into the beginning of the RTC and the VLPF in the RTC (**A + B**). It also showed that combining these two feedback locations offered greater performance over the individual locations tested in this study. However, the performance difference between BIV ’22 with inhibition at locations **A** and **B** and BIV ’22 with inhibition at location **D** was not found to be significant. Neurologically, both locations are be plausible; however, further studies would need to be undertaken to see if evidence for either one is supported biologically. The authors believe that BIV ’22 with inhibition at locations **A** and **B** may be the most logical location as all information is available at the start of the RTC, allowing for more a higher degree of sensitivity, whereas later in the model, decisions have been made and information reduced.

Tuning the temporal LPF in the MLI further improved performance by allowing a more accurate clutter/inhibition map to be generated for regions surrounding larger, more salient objects. The combination of the dynamically conditioned optic-flow inhibition at locations **A** and **B**, and tuning of the temporal LPF in the MLI provided a performance increase of nearly 19% relative to BIV ’22 with just inhibition at location **A**, 25% increase relative to BIV ’19, and 133% increase relative to BIV ’08.

The application of optic-flow inhibition to other algorithms also showed that their performance can be improved significantly, with LCM’s median AUROC performance increasing by a factor of 22.

Overall, this work has shown that the combination of local (as a measure of clutter) and global (as a measure of ego-motion) optic flow can be nonlinearly processed and used to suppress false positives when attempting to detect pixel sized targets in cluttered scenes from moving platforms.

## Supplementary Information

Below is the link to the electronic supplementary material.

Supplementary file 1 (pdf 5171 KB)
