# Supplementary material for: Exploration of motion inhibition for the suppression of false positives in biologically inspired small target detection algorithms from a moving platform
Source: Biol Cybern. 2022 Oct 28;116(5-6):661–85. doi: 10.1007/s00422-022-00950-9 (PMC9691501; doi:10.1007/s00422-022-00950-9)
Supplement: Supplementary file 1 — (pdf 5171 KB) [file 422_2022_950_MOESM1_ESM.pdf]

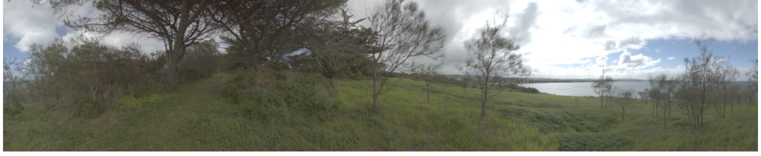
a) Bluff

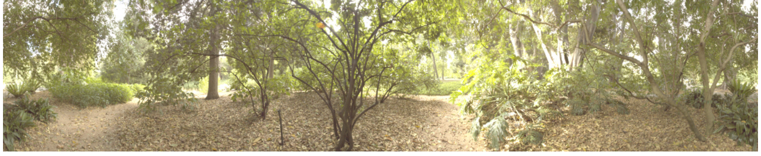
b) Botanic

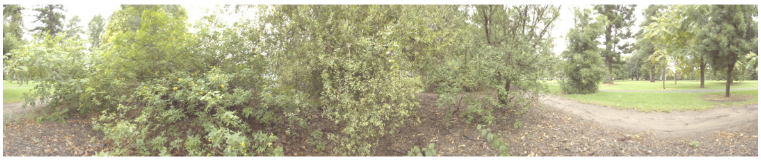
c) Bushes

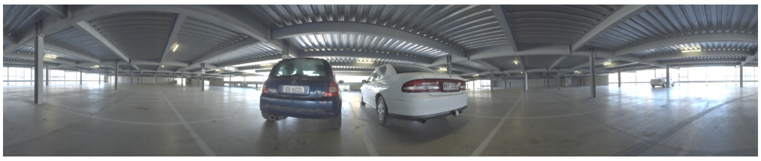
d) Car Park

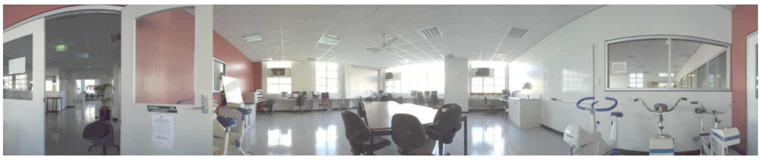
e) Classroom

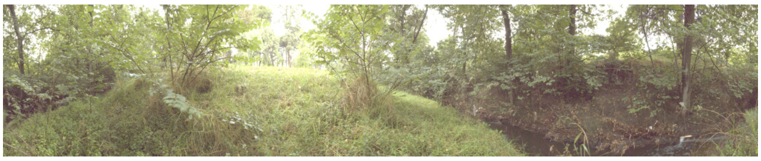
f) Creek Bed

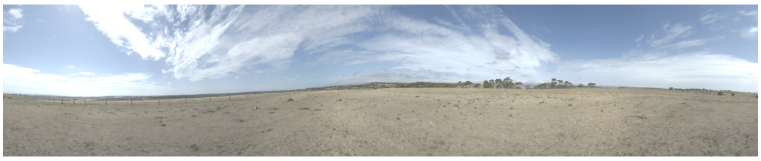
g) Field

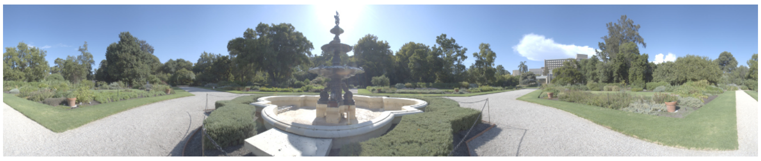
h) Fountain

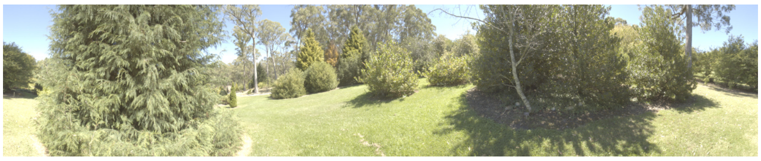
i) Hill

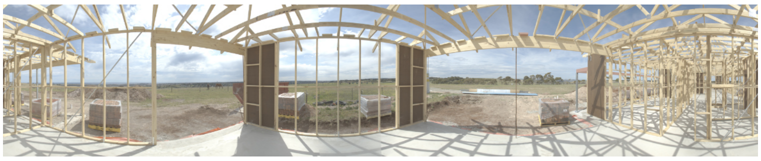
j) House

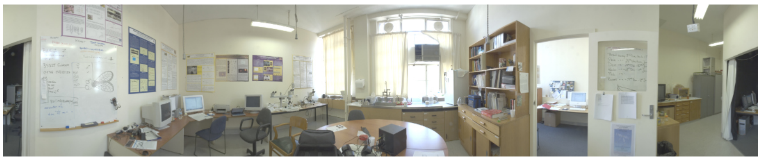
k) Lab

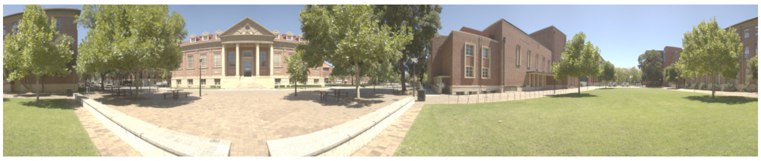
l) Library

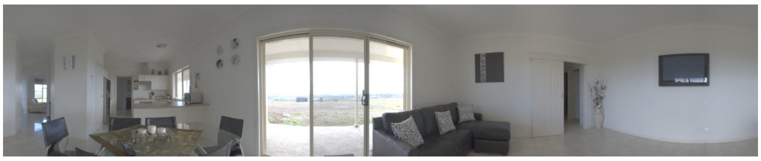
m) Lounge

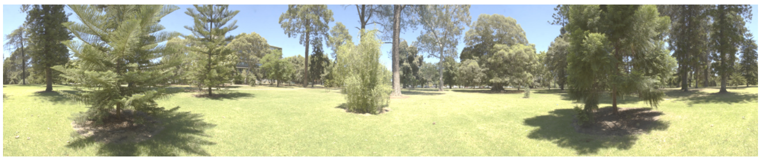
n) Outdoor

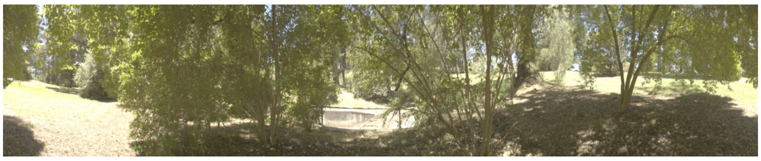
o) Park

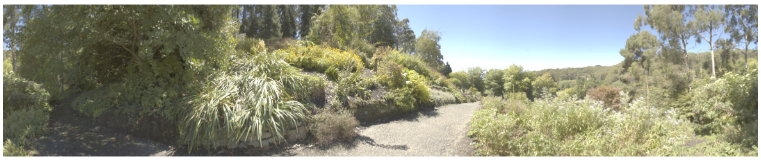
p) Rock Garden

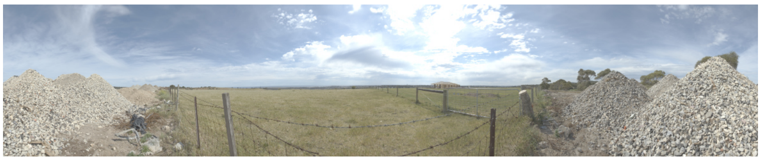
q) Rubble

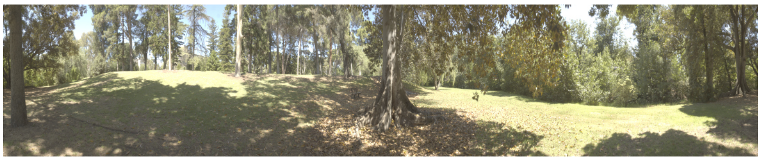
r) Shadow

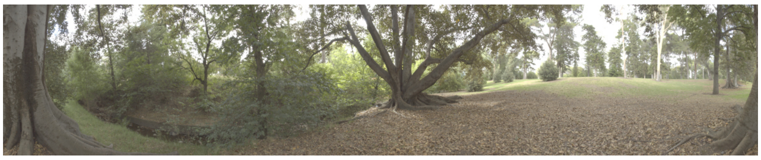
s) Tree

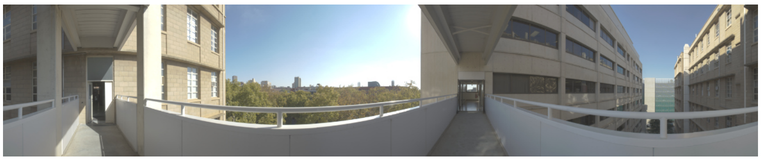
t) Walkway

The 20 HDR panormas used for backgrounds in this study.
